# Small RNA Expression Profiling Reveals hsa-miR-181d-5p Downregulation Associated With TNF-α Overexpression in Sjögren’s Syndrome Patients

**DOI:** 10.3389/fimmu.2022.870094

**Published:** 2022-04-01

**Authors:** Isabel Castro, Patricia Carvajal, Daniela Jara, Sergio Aguilera, Benjamín Heathcote, María-José Barrera, Víctor Aliaga-Tobar, Vinicius Maracaja-Coutinho, Ulises Urzúa, Andrew F. G. Quest, Sergio González, Claudio Molina, Marcela Hermoso, María-Julieta González

**Affiliations:** ^1^ Departamento de Tecnología Médica, Facultad de Medicina, Universidad de Chile, Santiago, Chile; ^2^ Programa de Biología Celular y Molecular, Instituto de Ciencias Biomédicas, Facultad de Medicina, Universidad de Chile, Santiago, Chile; ^3^ Departamento de Reumatología, Clínica Instituto de Diagnóstico Sociedad Anónima (Indisa), Santiago, Chile; ^4^ Facultad de Odontología, Universidad San Sebastián, Santiago, Chile; ^5^ Advanced Center for Chronic Diseases (ACCDiS), Facultad de Ciencias Químicas y Farmacéuticas, Universidad de Chile, Santiago, Chile; ^6^ Laboratorio de Bioingeniería, Instituto de Ciencias de la Ingeniería, Universidad de O’Higgins, Rancagua, Chile; ^7^ Departamento de Oncología Básico-Clínico, Facultad de Medicina, Universidad de Chile, Santiago, Chile; ^8^ Centro de Estudios en Ejercicio, Metabolismo y Cáncer (CEMC), Facultad de Medicina, Universidad de Chile, Santiago, Chile; ^9^ Escuela de Odontología, Facultad de Medicina y Ciencias de la Salud, Universidad Mayor, Santiago, Chile; ^10^ Programa de Inmunología, Instituto de Ciencias Biomédicas, Facultad de Medicina, Universidad de Chile, Santiago, Chile

**Keywords:** Sjögren’s syndrome, next generation sequencing, microRNA, hsa-miR-181d-5p, TNF-α

## Abstract

MicroRNAs (miRNAs) are small non-coding RNAs (sRNA), that alter gene expression by binding to target messenger RNAs (mRNAs) and repressing translation. Dysregulated miRNA expression has been implicated in the pathogenesis of autoimmune diseases such as Sjögren’s syndrome (SS). The aim of this study was to characterize the global profile of sRNAs in labial salivary glands (LSG) from SS-patients and to validate potential miRNA candidates implicated in glandular inflammation. LSG from 21 SS-patients and 9 sicca controls were analyzed. A global next generation sequencing (NGS)-based sRNA profiling approach was employed to identify direct targets whereby differentially expressed miRNAs were predicted using bioinformatics tools. miRNA levels were validated by TaqMan and target mRNA levels were determined by quantitative real-time PCR. We also performed *in vitro* assays using recombinant TNF-α. NGS shows that ~30% of sRNAs were miRNAs. In comparison with samples from sicca controls, four miRNAs were found differentially expressed in LSG from SS-patients with low focus score (LFS) and 18 from SS-patients with high focus score (HFS). The miRNA with the most significant changes identified by NGS was hsa-miR-181d-5p and downregulation was confirmed by TaqMan analysis. Levels of TNF-α mRNA, a direct target of hsa-miR-181d-5p, were significantly increased and negatively correlated with hsa-miR-181d-5p presence. Moreover, positive correlations between TNF-α transcript levels, focus score, ESSDAI, and autoantibody levels were also detected. Furthermore, TNF-α stimulation decreased hsa-miR-181d-5p levels *in vitro*. Downregulation of hsa-miR-181d-5p in LSG from SS-patients could contribute to the glandular pro-inflammatory environment by deregulation of its direct target TNF-α. Further dissection of the pathophysiological mechanisms underlying the hsa-miR-181d-5p-mediated action in inflammatory conditions could be useful to evaluate the benefits of increasing hsa-miR-181d-5p levels for restoration of salivary gland epithelial cell architecture and function.

## Introduction

The protein coding portion of the genome represents only a small percentile of the total genome and, importantly, the non-coding genome, produces many functional non-coding RNAs (ncRNAs) with multiple roles in diverse physiological and pathological conditions (1–7). Several forms of ncRNAs have been described, including long ncRNAs (> 200 nucleotides in length), small nuclear RNAs (snRNAs), small nucleolar RNA (snoRNAs), small interfering RNAs (siRNAs), PIWI-interacting RNAs, circular RNAs (circRNAs) and microRNAs (miRNAs), among others, whereby miRNAs the most widely studied RNA species (3, 7). MiRNAs are evolutionary conserved, single-stranded, small non-coding RNA (sRNA) molecules, ranging in size from 17 to 25 nucleotides (3, 8). They affect gene expression functioning as guide molecules in post-transcriptional gene silencing by binding to the 3’ untranslated region (UTR) of their target messenger RNAs (mRNAs), causing mRNA decay and blocking translation (3, 8). Expression analyses have shown that miRNAs are precisely regulated in a spatially and temporally specific manner in many biological processes. Thus, altering their expression and function can lead to aberrant biological consequences or pathological phenotypes (9, 10). Indeed, the dysregulated expression of miRNAs plays crucial roles in the pathogenesis of autoimmune diseases due to their modulatory effects in the immune system (11–14).

Sjögren’s syndrome (SS) is a systemic, chronic and inflammatory immune-mediated exocrinopathy, known as autoimmune epitheliitis, characterized by reduced secretory function, mainly of lacrimal and salivary glands (15). As with many autoimmune diseases, there is a lack of understanding of the etiologic events that lead to SS. For many years, the loss of immune tolerance in this pathology has been associated with the action of the immune system cells, such as B and T cells. However, a growing body of evidence has uncovered the importance of salivary gland cells and how alterations in epithelial homeostasis could be the initiating event, leading to secretory dysfunction, breakdown of immune tolerance, inflammation and tissue damage observed in SS (15–18). Here, it is important to emphasize that many of these changes have been observed to occur in a manner independent of the proximity of inflammatory cells (18). SS-patients have increased systemic and glandular levels of pro-inflammatory cytokines, synthesized by both immune and epithelial cells (19, 20). Of note, particularly TNF-α plays an important role in determining integrity of salivary acini architecture and, as a consequence, in epithelial function (17, 21–23).

TNF-α is thought to contribute to the development of autoimmune diseases by promoting expression of other proinflammatory cytokines, recruitment of inflammatory cells and damage of organs (24, 25). In labial salivary glands (LSG) from SS-patients, TNF-α is secreted by epithelial cells, CD4^+^ T lymphocytes and mononuclear cells infiltrating the glands (26). Although anti-TNF-α antibody therapy has not been shown to be clinically effective in SS-patients (27), TNFα plays a major role in the loss of glandular homeostasis and secretory dysfunction (17, 21–23). Tight junctions (TJ) are master regulators of cell polarity and additionally modulate the paracellular flow of ions and water (21). Differences in TJ protein levels and redistribution from the apical domain to the basolateral plasma membrane were detected in LSG SS-patients (21). Isolated acini from control subjects stimulated with TNF-α reproduced these alterations *in vitro* (21). A very important point that deserves mentioning in this context is that altered cell polarity affects the correct function and localization of secretory machinery proteins, such as SNARE fusion receptors (28), and Rab3D involved in the recognition of mature secretory granules, determining thereby specificity of the fusion process (29). Altered distribution of these proteins together with formation of functional basolateral SNARE complexes are associated with ectopic basolateral exocytosis of salivary mucins towards the extracellular matrix (ECM) (28). Ectopically secreted mucins activate pro-inflammatory signaling events *via* the Toll-like receptor 4, contributing to the initiation and perpetuation of local autoimmune responses (30). TNF-α stimulation of 3D-acini alters the directionality of the secretory process by increasing basolateral exocytic events (22). Moreover, in 3D-acini stimulated with TNF-α, similar ER stress events to those observed in LSG from SS-patients are observed (17, 23), including activation of the ATF6α pathway, increased nuclear translocation of ATF6f (17) as well as overexpression of SEL1L and EDEM1 (23), two key components of the ER associated protein degradation (ERAD) machinery. TNF-α incubation also induces MUC1 overexpression and accumulation in the ER (23). In addition to the morpho-functional alterations described here, TNF-α promotes epigenetic changes in epithelial cells, leading to the global DNA hypomethylation and increased TET-2 levels that have been observed in LSG from SS-patients (31).

In SS, potential clinical implications of miRNAs in autoantigen expression and autoantibody production (12, 32), as well as aberrant immune cell regulation and cytokine production (33, 34) have been reported. For example, in CD14^+^ monocytes freshly isolated from peripheral blood of SS-patients, several miRNAs are differentially upregulated, preferentially targeting TGF-β signaling pathway components, such as SMAD4, whose expression is repressed in a subpopulation of SS-patients. Alternatively, for pro-inflammatory IL-12 and NF-κB-dependent pathways, no evidence is available implicating upregulation of SS-monocyte miRNAs. TGF-β signaling is critical for maintaining a regulatory immune phenotype and controlling autoimmune progression, suggesting a preponderant role for this factor in promoting the development of a pro-inflammatory phenotype in SS-monocytes (34). In addition, mechanistic studies have shown the involvement of specific miRNAs in glandular dysfunction. For example, miR-142-3p is overexpressed in the salivary glands of SS-patients. This miRNA targets the 3’-UTR of the sarco(endo)plasmic reticulum Ca^2+^ATPase 2b (SERCA2B) pump, the ryanodine receptor 2 (RyR2), and adenylate cyclase 9 (AC9), key components of Ca^2+^ signaling involved in salivary gland secretion. miR-142-3p -containing exosomes from activated T cells impair epithelial cell function by altering Ca^2+^ flux, cAMP production, as well as protein secretion by decreasing SERCA2B, RyR2 and AC9 expression. Taken together, these observations implicate miR-142-3p as a key driver of the secretory dysfunction in SS-patients (35). Moreover, miRNA expression analyses have suggested their potential utility as biomarkers (33, 36–38). In this context, similar levels of miR200b-5p were found in minor salivary glands from SS-patients and sicca controls; however, levels of this miRNA were significantly reduced long before clinical onset of non-Hodgkin’s lymphoma (NHL) in SS-patients who will develop or have NHL, permitting therefore distinguishing between such patients and those without lymphoma or non-SS sialadenitis. Thus, low levels of miR200b-5p in minor salivary glands represent a predictive factor for the development of SS-associated NHL (39).

Various techniques for miRNA analysis have been applied in previous studies. More recently next-generation sequencing (NGS) has become the method of choice, by permitting compiling and highlighting differentially expressed miRNA and their potential implications in SS pathogenesis. In this study, we used NGS to characterize the global profile of sRNAs present in LSG from SS-patients and controls. Functional enrichment analysis identified several differentially expressed miRNAs involved in signaling pathways previously reported to be altered in SS-patients. From the sRNA NGS differential expression analysis, we selected the hsa-miR-181d-5p for further analysis, given that it represented the top differentially expressed miRNA in LSG from SS-patients when comparing with controls.

In the human genome, the miR-181 family is composed of six different 5p mature forms that share the same seed region and therefore are predicted to recognize a similar set of target genes (40). Aberrant expression of miR-181 family members has been described in neurodegenerative disorders (Alzheimer and Parkinson’s Disease), in solid tumors (hepatocellular, colorectal, non-small cell lung, pancreatic, ovarian, prostate, breast, brain, and oral cancers) and in hematological malignancies (leukemia, Burkitt lymphoma and multiple myeloma) (40). In hematological cancers, members of the miR-181 family act as either tumor suppressors or oncomiRs, by regulating the differentiation and development of immune cells (40). There are currently no studies concerning the role of miR-181 in the development of hematological malignancies in SS. On the other hand, growing evidence supports an important role for the miR-181 family in inflammation *via* the control of key signaling pathways (41–43). Interestingly, a relevant target of hsa-miR-181d-5p is TNF-α, one of the main pro-inflammatory cytokines overexpressed in both salivary glands and serum from SS-patients (19, 20, 26). Additionally, as was mentioned above, presence of this cytokine correlates strongly with alterations in salivary glandular architecture and function. For this reason, we focused our attention here on this particular miRNA.

In this context, using a global NGS-based sRNA profiling approach followed by validation with Taqman assays, we demonstrated that hsa-miR-181d-5p is downregulated in LSG from SS-patients and these results correlated with elevated glandular expression levels of TNF-α and the clinical features typical of SS-patients. Bearing this in mind, future studies increasing hsa-miR-181d-5p levels could contribute to improve salivary gland epithelial cell architecture and function.

## Material and Methods

### Patients With Sjögren’s Syndrome and Controls

The study group included 21 patients diagnosed with primary SS, based on the 2016 ACR/EULAR Classification Criteria (44). All these patients have a positive LSG biopsy. Patients presented the first manifestations that precedes the diagnosis suggestive of SS at least 2 to 3 years before consulting a rheumatologist (n=21, range [3 months-5 years], mean= 2.6 years, median= 3 years). These manifestations include exocrine manifestations like dry eye, dry mouth, and parotid swelling. At the time of biopsy, most of the SS-patients (14/21) were not taking drugs. In the case of the SS-patients who were taking drugs (7/21), it was suspended one month prior to the biopsy. These drugs correspond mainly to corticosteroids and/or disease-modifying antirheumatic drugs (DMARDs) such as methotrexate and hydroxychloroquine (for more details see **Supplementary Table S1**). Once diagnosed with primary SS, they have continued treatment to this day.

The sicca control group included 9 subjects with oral dryness symptoms, who did not fulfill the primary SS classification criteria, did not suffer systemic diseases and LSG biopsy analysis was informed as normal or with mild diffuse chronic sialadenitis. **Table 1** summarizes the demographic, serological, and histological characteristics of SS-patients and sicca controls. All the individuals signed an informed consent according to the Declaration of Helsinki and the study was approved by the Ethics Committee of the Facultad de Medicina, Universidad de Chile (N° 010-2016).

**Table 1:** Demographic and serological characteristics of SS-patients and control subjects.

	sRNA Next generation sequencing			Taqman/RT-qPCR assays		
	Sicca controls	SS-patients (low focus score)	SS-patients (high focus score)	Sicca controls	SS-patients (low focus score)	SS-patients (high focus score)
N	3	3	3	6	10	5
Gender, female/male	2/1	3/0	3/0	6/0	10/0	5/0
Age, x̄ (range), years	44 (40–52)	58 (51-68)	42 (34-55)	40 (30-49)	34 (21-51)	34 (17-52)
FS^#^, mean (range)	0 (0-0)	2.3 (1-3)	6.3 (4-8)	0 (0-0)	2 (1-3)	5.2 (4-7)
USWSF, mean (range)	5 (3-7.5)	1 (0-1.5)	1.2 (0-1.8)	3.1 (1.2-4.5)	1.9 (0.6-3.9)	0.15 (0-0.6)
Schirmer’s test ≤ 5 mm/5 min in at least one eye N° (%)	0 (0%)	3 (100%)	3 (100%)	1 (17%)	5 (50%)	3 (60%)
Anti-Ro (+) N° (%)	0 (0%)	3 (100%)	3 (100%)	0 (0%)	10 (100%)	5 (100%)
Anti-La (+) N° (%)	0 (0%)	2 (67%)	2 (67%)	0 (0%)	4 (40%)	3 (60%)
ANA (+) N° (%)	0 (0%)	3 (100%)	3 (100%)	2 (33.3%)	10 (100%)	5 (100%)
RF (+) N° (%)	0 (0%)	2 (67%)	1 (33%)	0 (0%)	4 (40%)	5 (100%)
ESSDAI, median (IQR 25-75)	–	10 (7,5-12)	10 (8-16)	–	6 (1-11)	17 (9-18)

USWSF, Unstimulated whole salivary flow (mL/15 min); ^#^FS, Focus score (number of foci/4 mm^2^ of tissue); ANA, antinuclear antibodies; RF, Rheumatoid factor; ESSDAI, EULAR Sjögren syndrome disease activity index; IQR, interquartile range.

### Biopsies of Labial Salivary Glands

LSG biopsies from SS-patients and sicca controls were performed according to Daniels et al. (45). Once obtained, some of the glands were used for diagnostic purposes and the rest were immediately frozen in liquid nitrogen.

### Small RNA Next Generation Sequencing

**Figure 1:**
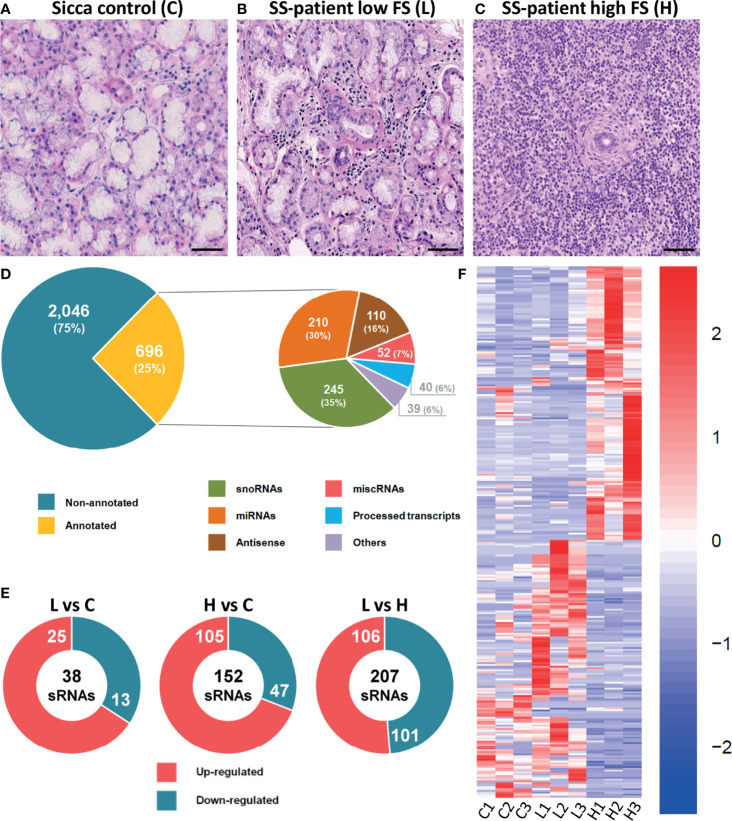
Global profile of small RNAs detected in LSG using NGS. **(A–C)** Histology of LSG from a representative sicca control, SS-patients with low focus score (L), and SS-patients with high focus score (H). **(D)** Total number of annotated and non-annotated sRNAs (left) and subgroups of snoRNAs, miRNAs, antisense, miscRNAs, processed transcripts and others are presented (right). **(E)** Number of sRNAs up-regulated or down-regulated in LSG from SS-patients with low (L) or high (H) focus score compared to sicca controls **(C)**. **(F)** Heat map of differentially expressed sRNAs in LSG from controls and SS-patients with low and high focus score. The rows of the heat map represent sRNAs, while the columns represent different LSG samples. The filter used was Log2FC=|2| and p-value ≤ 0.05.

Total RNA extraction enriched in sRNAs was performed using the miRNeasy mini kit (QIAGEN Sciences, Maryland, USA) from 3 LSG sicca control samples (C), 3 LSG from SS-patients with abundant parenchyma and low (L) focus score (FS <4) and 3 LSG from SS-patients with very scarce parenchyma, which has been mostly replaced by inflammatory cells [high (H) focus score (≥4)] (**Table 1**). Representative histology images of LSG from each group are shown in **Figure 1** and **Supplementary Figure S1**. To remove residual amounts of DNA contamination in isolated RNA, on-column DNase I digestion with RNase-free DNase (QIAGEN Sciences, Maryland, USA) was performed. The extracted RNAs were quantified by QuantiFluor fluorometry (Promega, WI, USA) and the quality and integrity control of the small RNA fragments were determined using the Agilent 2100 Bioanalyzer System (**Supplementary Figure S2**). Starting from 1 μg of total RNA from each sample, the cDNA libraries were prepared using the TruSeq SmallRNA^®^ kit (Illumina, CA, USA). Briefly, 3’ and 5’ adapters were sequentially ligated to each end of the miRNAs and other sRNAs, followed by a reverse transcription reaction. The cDNA was then amplified by PCR with 2 primers annealing to the ends of the adapters. The PCR product was selected according to size by using gel purification and validated using the Agilent 2100 Bioanalyzer System (**Supplementary Figure S3**). Illumina NGS was performed at Genoma Mayor, Universidad Mayor, Chile, on an Illumina MiSeq sequencer for 36 cycles of single-end sequencing.

### Bioinformatic Analysis

The raw reads generated by sequencing were trimmed using the following parameters: clipping the first ten bases in each side of read using Trimmomatic version 0.36 (46) considering a quality threshold of Q<30, with a five base sliding window quality filtering, eliminating thus reads for which the average quality of a window fell below Q<30. The reconstruction of small RNA transcripts, their annotation and estimation of expression levels was performed as previously described (47, 48). The steps involved were as follows: Only reads greater than 15 nt after trimming were mapped to the human genome (GRCh37/hg19) using Bowtie version 1.0.0 (48, 49), allowing only one mismatch and suppressing reads with more than 10 alignments. The SAM files generated were converted into BED format in order to use them for coordinates-bases assembly through BEDtools (50). Reads of each assembled sRNA transcript were recovered and used to estimate expression levels. Those transcriptional fragments with less than 50 reads in total were removed. The functional annotation of each sRNA class was recovered through cross-referencing genomic coordinates of assembled sRNA transcripts against the coordinates of the microRNAs from miRBase version 21 (51) and the ncRNAs from Ensembl version 92 (51, 52) using BEDtools intersectBed (50), considering an overlap of 50% between the sRNA transcripts and the reference ncRNAs. sRNA sequencing data is available at the BioProject database (ID: PRJNA685189). Differential expression analysis was performed using DESeq2 version 1.28.0 (53). Transcripts were considered as statistically differentially expressed when presenting a Log2FC ≥|2| and p-value ≤0.05. Gene ontology (GO) and overrepresentation analysis of target genes of each differentially expressed miRNA was performed using EnrichR (http://amp.pharm.mssm.edu/Enrichr/) (54, 55) and Webgestalt (WEB-based GEne SeT AnaLysis Toolkit, http://www.webgestalt.org/) (56). mirDIP (57) was used for target prediction and DIANA-miRPath v3. 0 (58) was used for miRNA pathway analysis.

### Taqman Assays

Total RNA extraction enriched in small RNAs was performed as described above from LSG from 6 sicca controls, 10 SS-patients with low focus score (LFS) and 5 SS-patients with high focus score (HFS) (**Table 1**). Forty nanograms of total RNA was reverse transcribed into cDNA using the Taqman™ MicroRNA Reverse Transcription Kit (Applied Biosystems, CA, USA) and Taqman™ MicroRNA assays (Applied Biosystems, CA, USA) that include specific RT probes for hsa-miR-181d-5p. To determine the expression levels of hsa-miR-181d-5p Taqman™ Universal Master Mix II, no UNG (Applied Biosystems, CA, USA) and Taqman™ MicroRNA assays that include specific PCR probes for hsa-miR-181d-5p were used. qPCR reactions were performed at least in triplicate using a MxPro 3000 thermocycler (Stratagene). miRNA levels were normalized to those of the U6, and for calculating relative expression levels the efficiency-calibrated model was used (59).

### Quantitative Real Time-PCR

Total RNA from LSG was extracted from 6 sicca controls, 10 SS-patients with low focus score (LFS) and 5 SS-patients with high focus score (HFS) (**Table 1**), using the miRNeasy mini kit (QIAGEN Sciences, Maryland, USA) as described above. One µg of total RNA was reverse transcribed with oligo (dT), random primers and the Superscript II enzyme (Invitrogen by Thermo Fisher Scientific, USA). Specific primers for TNF-α and h18S genes were designed with the AmplifiX 1.4 software (**Supplementary Table S2**). For qPCR reactions, the 5X Hot FirePol EvaGreen qPCR Mix Plus (Solis BioDyne, Estonia) was used. Relative quantification of TNF-α transcript was accomplished by comparative Ct analysis, using the efficiency-calibrated model and, h18S as a housekeeping gene (59).

### Cell Culture and TNF-α Stimulation

Human submandibular gland (HSG) cells were cultured as previously described (30, 60) and incubated with or without 1 ng/mL human recombinant TNF-α (BioLegend, CA, USA) in serum-free medium for 24 h and subsequently lysed to isolate RNA.

### Statistical Analysis

Mean values in LFS or HFS SS-patients and sicca control groups, as well as in HSG cells incubated with or without TNF-α were compared using the Mann-Whitney test. Spearman rank correlation analysis was also performed whereby P values less than 0.05 considered significant.

## Results

### Expression Profiling Using sRNA NGS

NGS is a powerful method enabling the analysis of both sRNA expression and sequencing, as well as identifying different forms of sRNA molecules. Here, by NGS we analyzed the global expression profile of sRNAs isolated from LSG of SS-patients with LFS, SS-patients with HFS, and sicca controls. Representative histology images of each group are shown in **Figures 1A–C** and **Supplementary Figure S1**. After selecting high quality reads, sequences were mapped to the reference genome allowing us to determine that 25% were annotated sequences (**Figure 1D**), of which 30% were miRNAs, 35% snoRNAs, 16% antisense, 7% miscoding RNAs (miscRNAs), 6% processed transcripts, and 6% others (**Figure 1D**). The differential expression analysis showed that 25 sRNAs were up-regulated and 13 down-regulated in LSG from SS-patients with LFS compared with sicca controls (**Figure 1E**). In samples from SS-patients with HFS, 105 sRNAs were up-regulated and 47 down-regulated, with respect to sicca controls (**Figure 1E**), and 106 sRNAs up-regulated and 101 down-regulated in SS-patients with LFS, compared with those with HFS (**Figure 1E**). The overall levels of sRNAs differed considerably between the three groups (**Figure 1F**).

### Identification of Differentially Expressed miRNAs in LSG of SS-Patients and Sicca Controls

**Figure 2:**
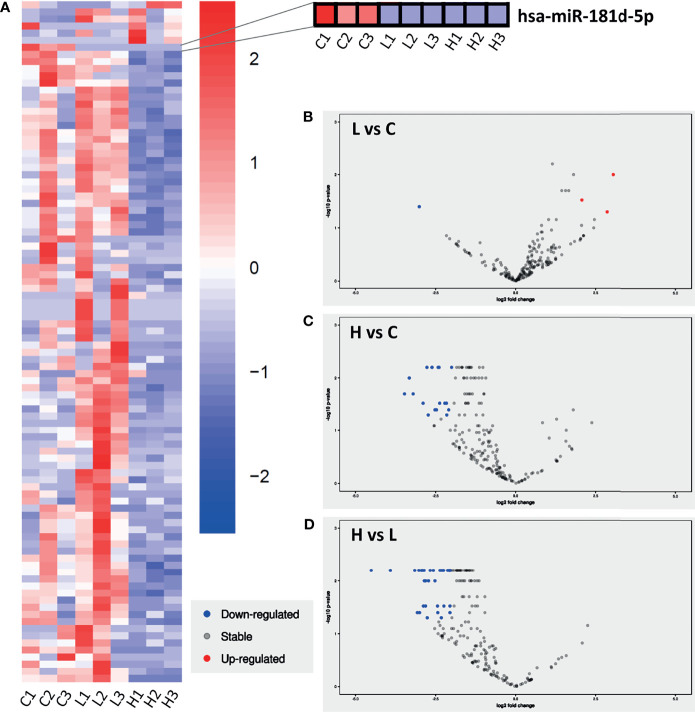
Expression profile of miRNAs found in LSG from SS-patients and sicca controls using NGS. **(A)** Heat map of differentially expressed miRNAs in LSG from sicca controls (C) and SS-patients with low (L) and high (H) focus score (FS). The rows of the heat map represent miRNAs, while the columns represent different LSG samples. A zoom to the hsa-miR-181d-5p is displayed. **(B-D)** Volcano plots of miRNAs down-regulated (blue dots), up-regulated (red dots) or stable (grey dots) found in LSG from SS-patients with low (L) FS, compared to sicca controls **(B)**; high (H) FS, compared to sicca controls **(C)**; and low (L) FS, compared to high (H) FS **(D)**. The filter used was Log2FC=|2| and p-value ≤ 0.05.

The purpose of any miRNA profiling study is to examine the mechanism by which specific miRNAs affect the cellular function. miRNA binding sites are usually located in the 3’ UTR region of the target mRNA, where a partial complementary base pairing of miRNA seed region target a mRNA, typically inducing translational repression (8). Moreover, a target mRNA can have multiple miRNA binding sites, as well as a miRNA having diverse mRNA targets (11). In this study, miRNAs were clustered using normalized read counts detected in LSG of SS-patients with LFS or HFS, and sicca controls (**Figure 2A**). From the total miRNAs detected in the NGS analysis, only 4 were found to be differentially expressed in LSG from SS-patients with LFS compared to sicca controls, 3 of which were up-regulated and 1 down-regulated (**Figure 2B** and **Table 2**). In samples from SS-patients with HFS, 18 miRNAs were down-regulated compared to sicca controls (**Figure 2C** and **Table 2**). Among SS-patients, 32 miRNAs were down-regulated in those with HFS compared to LFS (**Figure 2D** and **Table 2**).

**Table 2:** miRNAs differentially expressed in labial salivary glands from control subjects and SS-patients with low or high focus score.

Low focus score vs Controls			High focus score vs Low focus score		
miRbase annotation	log2FC	p-value	miRbase annotation	log2FC	p-value
**hsa-miR-181d-5p**	**-3,01**	**0,037**	hsa-miR-136-3p	-4,51	0,000
hsa-miR-30e-3p	2,07	0,025	hsa-miR-345-5p	-2,76	0,049
hsa-miR-493-5p	2,86	0,050	hsa-miR-582-5p	-3,91	0,003
hsa-miR-769-5p	3,05	0,014	hsa-miR-23b-3p	-3,02	0,001
			hsa-miR-375-3p	-2,05	0,002
			hsa-miR-148a-5p	-2,67	0,000
			hsa-miR-200c-3p	-2,07	0,000
**High focus score vs Controls**			hsa-miR-30a-3p	-2,52	0,012
**miRbase annotation**	**log2FC**	**p-value**	hsa-miR-125b-2-3p	-2,94	0,002
**hsa-miR-181d-5p**	**-3,47**	**0,016**	hsa-miR-183-5p	-2,72	0,012
hsa-miR-136-3p	-3,32	0,008	hsa-miR-199a-5p	-2,81	0,005
hsa-miR-345-5p	-3,20	0,020	hsa-miR-93-5p	-2,32	0,047
hsa-miR-582-5p	-2,89	0,030	hsa-miR-320a-3p	-2,81	0,028
hsa-miR-23b-3p	-2,77	0,002	hsa-miR-320c	-3,00	0,037
hsa-miR-424-5p	-2,73	0,055	hsa-miR-409-3p	-2,44	0,028
hsa-miR-335-5p	-2,61	0,003	hsa-miR-381-3p	-2,24	0,029
hsa-miR-375-3p	-2,61	0,000	hsa-miR-125b-5p	-2,08	0,001
hsa-miR-24-3p	-2,52	0,041	hsa-miR-411-5p	-2,86	0,000
hsa-miR-660-5p	-2,47	0,044	hsa-miR-199a-3p	-2,25	0,001
hsa-miR-148a-5p	-2,42	0,001	hsa-miR-100-5p	-2,57	0,000
hsa-miR-200c-3p	-2,39	0,000	hsa-miR-30e-3p	-3,15	0,001
hsa-miR-23a-3p	-2,37	0,028	hsa-miR-340-5p	-2,05	0,026
hsa-miR-30a-3p	-2,21	0,025	hsa-miR-145-5p	-2,12	0,003
hsa-miR-125b-2-3p	-2,16	0,026	hsa-miR-24-3p	-2,05	0,039
hsa-miR-183-5p	-2,15	0,048	hsa-miR-3074-5p	-2,05	0,039
hsa-miR-199a-5p	-2,09	0,038	hsa-miR-181a-2-3p	-2,30	0,001
			hsa-miR-497-5p	-2,88	0,030
			hsa-miR-374a-5p	-2,43	0,040
			hsa-miR-493-5p	-3,07	0,036
			hsa-miR-769-5p	-2,85	0,014

FC, folding change; FS, focus score. The top differentially expressed miRNA found in SS-patients compared to sicca controls is highligted with bold text.

### Functional Enrichment Analysis of Differentially Expressed miRNAs

We performed a functional enrichment analysis using DIANA-miRPath v3.0 (58) to identify signaling pathways regulated by miRNAs differentially expressed in SS-patients with LFS compared to sicca controls (**Supplementary Table S3**), in SS-patients with HFS compared to sicca controls (**Supplementary Table 4**) and SS-patients with LFS compared to SS-patients with HFS (**Supplementary Table S5**). In this section we describe just a few examples of relevant target genes that have been previously found to be overexpressed or downregulated in LSG of SS-patients.

In LSG from SS-patients with LFS compared to sicca controls, we found 3 miRNAs differentially expressed (hsa-miR-769-5p, hsa-miR-493-5p and hsa-miR-181d-5p), implicated in regulating 4 genes encoding for glycosyltransferases of the Golgi apparatus involved in mucin O-Glycan biosynthesis (POC1B-GALNT4, GALNT4, GALNT10 and C1GALT1) (**Supplementary Table S3**). This observation (**Table 2**) is in agreement with a previous report connecting this miRNA to the downregulation of GALNT4 (61), a member of the GalNAc-T family of enzymes which initiates O-glycosylation of mucins in epithelial cells, a process that is altered in SS-patients (23, 61). The downregulation on hsa-miR-769-5p observed in LSG from SS-patients with HFS compared to SS-patients with LFS (**Table 2**) may be a consequence of the loss of the epithelial component and its replacement by inflammatory cells in the LSG from SS-patients with HFS. In LSG from SS-patients with LFS compared to sicca controls we detected 4 differentially expressed miRNAs targeting 24 genes involved in protein processing in the endoplasmic reticulum (**Supplementary Table S3**). This canonical pathway also identifies 95 target genes regulated by 16 miRNAs downregulated in SS-patients with HFS compared to sicca controls (**Supplementary Table S4**). Among these target genes, ATF6α, SEL1L, EDEM1 and DERL1 have been previously reported to be overexpressed in LSG from SS-patients (60). In LSG from SS-patients with LFS compared to sicca controls, we identified 4 differentially expressed miRNAs that regulate 13 genes involved in T cell receptor signaling pathway (**Supplementary Table S3**). Moreover, 14 miRNAs targeting 54 genes in this pathway were found to be downregulated in LSG from SS-patients with HFS compared to sicca controls (**Supplementary Table S4**). Some of these target genes encode pro-inflammatory cytokines, such as TNF-α and IFN-γ, and others anti-inflammatory cytokines, such as IL-10, and all of them are overexpressed in LSG from SS-patients (20).

In LSG from SS patients with HFS compared to sicca controls we found 15 downregulated miRNAs targeting 84 genes involved in actin cytoskeleton regulation, such as EZR (ezrin) (**Supplementary Table S4**), encoding a protein participating in the structural organization of the acinar cell apical pole that is overexpressed and mislocalized in LSG from SS-patients (62).

Functional enrichment analysis of differentially expressed miRNAs in LSG from SS-patients with LFS and SS-patients with HFS identified 32 mRNA encoded by genes involved in ECM-receptor interactions, which are predicted targets of 22 differentially expressed miRNAs (**Supplementary Table S5**). An example here being ITGA6 responsible for cell anchorage to the basal lamina of acinar cells, which is altered in SS-patients and causes hemidesmosome disorganization (63).

Together these results suggest that alterations in miRNAs levels in LSG from SS-patients could alter the expression of proteins essential for glandular homeostasis and secretory function.

### hsa-miR-181d-5p Is Downregulated in LSG From SS-patients Compared to Sicca Controls

**Figure 3:**
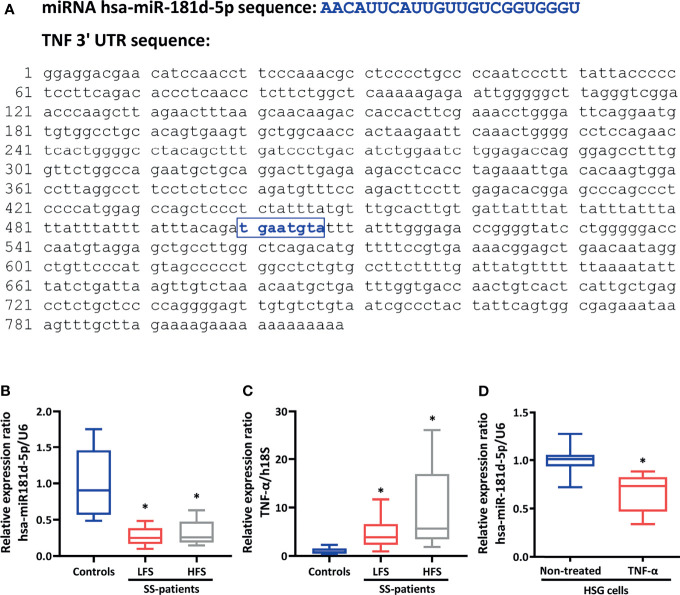
hsa-miR-181d-5p is downregulated and TNF-α is overexpressed in LSG from SS-patients. **(A)** The hsa-miR-181d-5p and TNF-α 3’-UTR sequences are shown. The box highlights the seed sequence. **(B)** Levels of hsa-miR-181d-5p in sicca controls (n=6) and SS-patients with low (n=10) and high focus score (n=5). U6 snRNA was used as a housekeeping. **(C)** TNF-α transcript levels in sicca controls (n=6) and SS-patients with low (n=10) and high focus score (n=5). h18S was used as a housekeeping gene. **(D)** Levels of hsa-miR-181d-5p in HSG cells stimulated with or without 1 ng/mL TNF-α. Data are representative of at least three independent measurements. (*) p-value ≤ 0.05 was considered significant.

After performing the comparative analysis of miRNA expression, we selected the hsa-miR-181d-5p, because it was the miRNA showing the greatest differences between SS-patients and sicca controls (**Table 2** and **Figure 3A**). In addition, TNF-α is targeted by hsa-miR-181d-5p and this cytokine has been linked to the loss of salivary gland epithelial cell architecture and function of LSG from SS-patients (17, 21–23). The NGS shows that hsa-miR-181d-5p levels were significantly lower in LSG from SS-patients with LFS (p=0.037) and HFS (p=0.016) compared to sicca controls (**Figure 2A** and **Table 2**). Using TaqMan miRNA assays in LSG from 15 SS-patients and 6 controls, we validated hsa-miR-181d-5p levels. We observed decreased expression in SS-patients with LFS (p=0.0002) and SS-patients with HFS (p=0.0173) compared to sicca controls (**Figure 3B**). To predict the target genes of hsa-miR-181d-5p, we used mirDIP analysis (57), which revealed a possible interaction between TFN-α and hsa-miR-181d-5p in 8 bioinformatic algorithms, including miRDB. There were 1408 possible target genes that are predicted to be regulated by hsa-miR-181d-5p (**Supplementary Table S6**). To test whether molecular functions or biological processes were associated with the hsa-miR-181d-5p, we performed a Gene ontology (GO) analysis. From the total number of target genes predicted to be regulated by hsa-miR-181d-5p, 14 genes are involved in the response to ER stress, previously reported to be overexpressed in LSG from SS-patients (60) (**Table 3**). Among them, SEL1L encodes for a protein involved in ubiquitin protein ligase binding and DERL1 for a protein that retrotranslocates misfolded or unfolded proteins from the ER lumen to the cytosol for proteasomal degradation. Both represent key components of the ERAD machinery, which is more active in SS-patients’ LSG (60). These results suggest that different transcriptional and post-transcriptional mechanisms are likely acting together to regulate the expression of molecules involved in glandular homeostasis in LSG from SS-patients.

**Table 3:** Gene Ontology (GO) analysis of biological processes associated with hsa-miR-181d-5p.

Geneset	Biological process	Reference genes in category	Number of Queried genes in category	Overlap Genes
GO:0036500	ATF6-mediated unfolded protein response	9	4	HSPA5; CALR; MBTPS2; HSP90B1
GO:1903798	Regulation of production of miRNAs involved in gene silencing by miRNA	18	5	DDX5; MAP2K1; LIN28A; ESR1; TNF
GO:2000637	Positive regulation of gene silencing by miRNA	27	6	DDX5; MAP2K1; LIN28A; FMR1; PUM1; TNRC6B
GO:0010586	miRNA metabolic process	20	5	TRIM71; LIN28B; LIN28A; TENT4B; RAN
GO:1902235	Regulation of endoplasmic reticulum stress-induced intrinsic apoptotic signaling pathway	22	5	PRKN; CREB3; BCL2L11; TXNDC12; RNF183
GO:0009312	Oligosaccharide biosynthetic process	23	5	B4GALT1; FUT9; B3GALT1; B3GALT5; ST6GALNAC5
GO:0045199	Maintenance of epithelial cell apical/basal polarity	9	3	PDCD6IP; PDZD11; LIN7A
GO:0034976	Response to endoplasmic reticulum stress	110	14	ERO1A; PRKN; HSPA5; SEL1L; DERL1; ATP2A2; PIK3R1; HSP90B1; BCL2L11; TMEM33; BCL2; HYOU1; RNF183; MBTPS2
GO:0043122	Regulation of I-kappaB kinase/NF-kappaB signaling	224	24	PRKN; MAP3K3; TFRC; PDPK1; ZFAND6; F2R; RORA; SIRT1; ZDHHC17; TNF; ESR1; MID2; FKBP1A; PPM1A; PPM1B; CD4; TNFSF10; REL; TAB3; TAB2; ZMYND11; TMEM9B; CARD11; TMED4
GO:0006515	Protein quality control for misfolded or incompletely synthesized proteins	25	5	ATXN3; KLHL15; CUL3; DERL1; FBXL17
GO:0044030	Regulation of DNA methylation	10	3	MECP2; KMT2A; DPPA3
GO:0043062	Extracellular structure organization	216	23	FBN2; MMP7; ITGA3; LAMA1; ITGA2; SERPINE1; DCN; TGFBR1; ADAMTSL1; ADAMTS5; ACAN; MMP14; VCAN; LOX; ADAMTS19; ADAMTS18; SPP1; ITGB8; COL6A3; ITGA6; TGFBI; RECK; ADAMTS6
GO:0031532	Actin cytoskeleton reorganization	63	9	MKLN1; RALA; EFS; DMTN; MINK1; S1PR1; TNIK; CDC42BPA; ANTXR1
GO:0071218	Cellular response to misfolded protein	20	4	ATXN3; KLHL15; CUL3; DERL1

Interestingly, 24 relevant targets of hsa-miR-181d-5p are involved in the regulation of I-κB kinase/NF-κB signaling (**Table 3**). Among these, we choose TNF-α, a multifunctional proinflammatory cytokine that belongs to the tumor necrosis factor superfamily and that may be a pathogenic factor in SS and other autoimmune diseases (24–26). The TNF-α 3’-UTR contains one hsa-miR-181d-5p binding site that has been reported to be crucial for TNF-α regulation (64) (**Figure 3A**).

### TNF-α Is Overexpressed and Associated With hsa-miR-181d-5p Downregulation and With Clinical Features in SS-Patients

TNF-α transcript levels were significantly increased in LSG from SS-patients with LFS (p= 0.0017) and SS-patients with HFS (p= 0.0087), compared to sicca controls (**Figure 3C**). Spearman’s analysis showed a negative correlation between hsa-miR-181d-5p levels and TNF-α transcript levels (R= -0.426; p= 0.0271) (**Supplementary Figure S4**). Positive correlations were found between TNF-α transcript levels and focus score (R= 0.74; p= <0.001), ESSDAI (R= 0.634; p= 0.003), RF (R= 0.529; p= 0.014), and ANA (R= 0.831; p= <0.001) autoantibody levels (**Supplementary Figure S5**). In addition, Spearman’s analysis revealed a negative correlation between hsa-miR-181d-5p levels and focus score (R= -0.529; p=0.014), ESSDAI (R= -0.628; p= 0.003), as well as ANA (R= -0.473; p= 0.041) autoantibody levels (**Supplementary Figure S4**).

### TNF-α Stimulation Downregulates hsa-miR-181d-5p in HSG Cells.

Increased levels of TNF-α have been detected in LSG, saliva and serum on SS-patients (19, 20, 26). To evaluate if the decreased levels of hsa-miR-181d-5p observed in SS-patients are associated with the inflammatory environment, we evaluated the effect of TNF-α on hsa-miR-181d-5p levels in HSG cells. A significant decrease in hsa-miR-181d-5p levels were detected in HSG cells stimulated with 1 ng/mL TNF-α for 24 h (**Figure 3D**).

## Discussion

Molecular mechanisms that drive SS onset are poorly understood; however, recent analysis of genomic and epigenomic changes has provided new insights into the understanding of disease pathogenesis (65). Altered salivary gland epithelia in SS-patients has been associated with changes in gene expression (65). ncRNAs have the capacity to modify protein levels by mechanisms independent of transcription, being relevant players in gene expression regulation (3). Here, we analyzed the global expression profile of sRNAs and found that about 25% of NGS reads were genome annotated sequences, of which about 30% were identified as miRNAs. Several of the differentially expressed miRNAs were predicted to target genes involved in protein processing in the ER and mucin O-glycan biosynthesis, both representing essential secretory processes that are altered in LSG from SS-patients (17). These findings confirm data from a previous report using miRNA array analysis that identified dysregulated miRNAs targeting glycosyltransferase and glycosidase genes involved in the mucin O-glycosylation in LSG from SS-patients (61). Other differentially expressed miRNAs in our NGS are predicted to target genes involved in the apical pole organization and cell-ECM interaction, again processes known to be dysregulated in LSG from SS-patients (62, 63) that affect the polarized epithelial cell architecture. Moreover, the mRNAs coding for these proteins were shown to be altered in a previous study using microarray analysis to compare cDNA isolated from acinar and ductal cells of LSG from SS-patients (66). The present findings highlight the potential importance of miRNA-mediated regulatory mechanisms in determining overexpression or downregulation of specific genes affecting the structure and function of LSG from SS-patients.

The hsa-miR-181d-5p was the top differentially expressed miRNA found in our NGS, and the downregulation of this miRNA in LSG from SS-patients with LFS or HFS was validated by TaqMan assays. Hsa-miR-181d-5p is a member of the highly conserved miR-181 family considered to be of increasing biomedical relevance (40). The members of the miRNA-181 family are both oncogenic and tumor-suppressive miRNAs, exerting their regulatory effects through modulating diverse signaling pathways, including PI3K/AKT, MAPK, TGF-β, Wnt, NF-κB, and Notch, among others (67). Expression of the miR-181 family is regulated by different mechanisms, such as DNA methylation, histone acetylation, TGF-β signaling and competing endogenous RNA (ceRNA) that sponge miRNA in shared binding sequences, which in turn sequester miRNAs from their targets (67). Growing evidence shows that miR-181 family members have a dual-role in inflammation, acting as both pro- and anti-inflammatory miRNAs, depending on the specific cell type, cell maturation status and external stimuli (41–43). In SS-patients, differential tissue-specific expression of miR-181a has been reported. While peripheral blood mononuclear cells from SS-patients showed elevated miR-181a levels (68), for LSGs significant downregulation of miR-181a was reported compared to non-SS sicca controls (69). miR-181 family members have been implicated in the development and function of immune cells, including roles in B and T cell differentiation, sensitivity, and selection (70, 71). In a mouse model of Systemic Lupus Erythematosus, low expression of miR-181 was associated with reduced differentiation of a rare subset of NK cells (72). In brain white matter from multiple sclerosis (MS)-patients and spinal cords of experimental autoimmune encephalomyelitis (EAE) mice, miR-181a and miR181-b levels were significantly decreased (73). miR-181a and miR181-b expression was also diminished in primary macrophages and lymphocytes following cell activation (73). Transfection of murine bone marrow-derived macrophages with miR-181a and miR181-b mimics diminished levels of TNF-α and IL6 transcripts (73). Increased levels of miR-181a and miR-181b also attenuate LPS-induced macrophage inflammatory responses and decrease the expression of M1-associated macrophage markers (73). These findings highlight the relevance of the anti-inflammatory role of miR-181a and miR-181b in autoimmune neuroinflammation (73).

A relevant target of hsa-miR-181d-5p is TNF-α, one of the main pro-inflammatory cytokines overexpressed in saliva, salivary glands and serum from SS-patients (19, 20, 26). The TNF-α 3’-UTR contains a miR-181 binding site (**Figure 3A**), which is highly conserved in mammals. It has been reported that mimics of miR-181a, miR-181b, miR-181c and miR-181d inhibited the luciferase activity of a TNF-α 3’-UTR reporter but had no effect when the miR-181 binding site was mutated (64). The miR-181d mimics also suppressed ouabain-induced TNF-α mRNA expression in A549 cells and TNF-α protein expression in human blood monocytes (64). Moreover, in a murine model of sepsis, administration of miR-181d mimics decreased CD4+ and CD8+ T cells in spleen and lymph nodes and reduced the TNF-α levels in serum. The same report showed that members of the miR-181 family act downstream of TLR4 signaling to induce TNF-α mRNA degradation (64). Here, downregulation of hsa-miR-181d-5p was inversely correlated with glandular TNF-α mRNA levels and other inflammatory signs, such as FS and levels of autoantibodies, suggesting an anti-inflammatory role of this miRNA in SS. Interestingly, HSG cells stimulated with TNF-α decreased its hsa-miR-181d-5p levels. However, further experiments are needed to shed light on the regulatory mechanism involved in this hsa-miR-181d-5p-TNF-α feedback loop.

Even though the number of samples analyzed by NGS is small, the validation of hsa-miR-181d-5p levels in 15 SS-patients was consistent, demonstrating its downregulation in LSG from SS-patients. Repressed expression of hsa-miR-181d-5p could contribute to the pro-inflammatory glandular environment by dysregulation of its direct target TNF-α. Further dissection of the pathophysiological mechanisms underlying hsa-miR-181d-5p-mediated action in inflammatory conditions could be useful to identify potential strategies permitting restoration of salivary gland epithelial cell architecture and function by modulating hsa-miR-181d-5p levels.

## Data Availability Statement

The datasets presented in this study can be found in online repositories. The names of the repository/repositories and accession number(s) can be found below: https://www.ncbi.nlm.nih.gov/bioproject/?term=PRJNA685189, BioProject PRJNA685189.

## Ethics Statement

The studies involving human participants were reviewed and approved by Ethics Committee of the Facultad de Medicina, Universidad de Chile. Written informed consent to participate in this study was provided by the participants’ legal guardian/next of kin.

## Author Contributions

IC, PC, and M-JG conceived the study. IC and PC conceived and designed the experiments while IC, PC, DJ, SA, BH, M-JB, VA-T, VM-C, UU, AQ, SG, CM and MH performed them. SA, SG, and CM were involved in clinical data collection. IC, PC and M-JG wrote the manuscript. All authors contributed to manuscript revision, read, and approved the submitted version.

## Funding

This work was supported by Fondecyt-Chile **(**1210055 to M**-**JG, SA, IC, CM, SG, M**-**JB**)**; Fondecyt-Chile **(**1160015 to M**-**JG, SA, IC, CM, SG**)**; Fondecyt-Chile **(**1210644 to AFGQ**)**; Enlace-VID Universidad de Chile **(**ENL04/20 to MJG**)**; FONDAP **(**15130011 to AQ, MH, VM**-**C**)**; Fondecyt-Iniciación **(**11201058 to M**-**JB**)**; PhD fellowship Conicyt-Chile to PC and DJ and Fondequip EQM170098.

## Conflict of Interest

The authors declare that the research was conducted in the absence of any commercial or financial relationships that could be construed as a potential conflict of interest.

## Publisher’s Note

All claims expressed in this article are solely those of the authors and do not necessarily represent those of their affiliated organizations, or those of the publisher, the editors and the reviewers. Any product that may be evaluated in this article, or claim that may be made by its manufacturer, is not guaranteed or endorsed by the publisher.
