# Supplementary material for: Small RNA Expression Profiling Reveals hsa-miR-181d-5p Downregulation Associated With TNF-α Overexpression in Sjögren’s Syndrome Patients
Source: Front Immunol. 2022 Apr 1;13:870094. doi: 10.3389/fimmu.2022.870094 (PMC9010469; doi:10.3389/fimmu.2022.870094)
Supplement: Supplementary file 1 [file DataSheet_1.doc]

**Supplementary Material**

**Small RNA expression profiling reveals hsa-miR-181d-5p downregulation associated with TNF-α overexpression in Sjögren's syndrome patients**

**Supplementary Table S1.** Number of SS-patients using at least one drug at the time of biopsy

| **Drug** | **SS-patients with low focus score** | **SS-patients with high focus score** |
| --- | --- | --- |
| **Prednisone** | 1/13 | 2/08 |
| **Hydroxychloroquine** | 1/13 | 1/08 |
| **Chloroquine** | 1/13 | 1/08 |
| **Methotrexate** | 0/13 | 1/08 |

**Supplementary Table S2.** Primers used for RT-qPCR assays

| **Gene** | **Accession number** | **Primer sequences** |
| --- | --- | --- |
| **TNF-α** | NM_000594.2 | F: 5’‐TCCGTGAAAACGGAGCTGAA‐3’  R: 5’‐AGGCTCAGCAATGAGTGACA‐3’ |
| **h18S** | NM_022551.2 | F: 5’‐GATATGCTCATGTGGTGTTG‐3’  R: 5’‐AATCTTCTTCAGTCGCTCCA‐3’ |

F: forward, R: reverse

**Supplementary Table S3. Functional enrichment analysis of differentially expressed miRNAs in LSG from SS patients with low focus score and sicca controls.**

| **Sicca controls vs SS-Patients with low focus score** | | |
| --- | --- | --- |
| **KEGG pathway** | **Examples of miRNAs** | **Examples of target mRNAs** |
| **Thyroid hormone signaling pathway** | **4 miRNAs**: hsa-miR-493-5p, hsa-miR-30e-3p, hsa-miR-769-5p, hsa-miR-181d-5p | **16 genes**: MED13L, MED13, ATP1B1, NCOA2, PIK3R1, PDPK1, ATP2A2, FOXO1, NCOA1, CREBBP |
| **Mucin type O-Glycan biosynthesis** | **3 miRNAs**: hsa-miR-769-5p, hsa-miR-493-5p, hsa-miR-181d-5p | **4 genes**: POC1B-GALNT4, GALNT4, GALNT10, C1GALT1 |
| **FoxO signaling pathway** | **4 miRNAs**: hsa-miR-181d-5p, hsa-miR-493-5p, hsa-miR-769-5p, hsa-miR-30e-3p | **27 genes**: TNFSF10, PIK3R3, PIK3R1, SOS1, BCL6, AKT3, FOXO3, PTEN, FOXO1, MAPK1, IL10, TGFBR2 |
| **Signaling pathways regulating pluripotency of stem cells** | **4 miRNAs**: hsa-miR-493-5p, hsa-miR-769-5p, hsa-miR-30e-3p, hsa-miR-181d-5p | **20 genes**: SMAD2, AKT3, WNT5A, AXIN1, ZFHX3, FZD3, PCGF5, FZD4, SKIL, PIK3R1, SOX2, KLF4 |
| **Wnt signaling pathway** | **4 miRNAs**: hsa-miR-493-5p, hsa-miR-181d-5p, hsa-miR-30e-3p, hsa-miR-769-5p | **25 genes**: FZD7, WNT5A, ROCK2, CUL1, AXIN1, FZD3, NLK, FZD4, WIF1, CREBBP |
| **Ubiquitin mediated proteolysis** | **4 miRNAs**: hsa-miR-30e-3p, hsa-miR-493-5p, hsa-miR-181d-5p, hsa-miR-769-5p | **19 genes**: UBE2Q2, UBE2E2, CUL1, KLHL13, CUL5, UBE3A, UBE2Q1, UBE2G1, PIAS2, PARK2, CDC27 |
| **Protein processing in endoplasmic reticulum** | **4 miRNAs**: hsa-miR-769-5p, hsa-miR-493-5p, hsa-miR-30e-3p, hsa-miR-181d-5p | **24 genes**: UBQLN1, UBE2E2, BCL2, CUL1, SEC63, MAN1A2, SEC62, UBE2D3, UBE2G1, DERL1 |
| **Ras signaling pathway** | **4 miRNAs**: hsa-miR-181d-5p, hsa-miR-30e-3p, hsa-miR-493-5p, hsa-miR-769-5p | **27 genes**: MAPK1, FGF13, EFNA3, EGFR, GNB1, SHC3, CALM2, PIK3R1, TBK1, PAK6, RAB5C, FGF7 |
| **mTOR signaling pathway** | **4 miRNAs**: hsa-miR-769-5p, hsa-miR-181d-5p, hsa-miR-493-5p, hsa-miR-30e-3p | **11 genes**: EIF4E, HIF1A, PIK3R1, PDPK1, PRKAA1, AKT3, MAPK1, DDIT4, PIK3R3, PTEN, RPS6KB1 |
| **PI3K-Akt signaling pathway** | **4 miRNAs**: hsa-miR-493-5p, hsa-miR-181d-5p, hsa-miR-30e-3p, hsa-miR-769-5p | **38 genes**: ITGB1, BCL2, EGFR, PPP2R5C, CREB1, EIF4E, PIK3R1, FOXO3, FGF7, PTEN, CREB5, ITGA8 |
| **cAMP signaling pathway** | **4 miRNAs**: hsa-miR-493-5p, hsa-miR-30e-3p, hsa-miR-769-5p, hsa-miR-181d-5p | **33 genes**: CAMK2D, CALM1, ROCK2, CALM2, PTGER3, CREB1, PIK3R3, NFKBIA, AKT3, MAPK1 |
| **T cell receptor signaling pathway** | **4 miRNAs**: hsa-miR-181d-5p, hsa-miR-30e-3p, hsa-miR-769-5p, hsa-miR-493-5p | **13 genes**: IL10, MAPK1, SOS1, PIK3R3, PPP3R1, PTPRC, PPP3CA, NFKBIA, PIK3R1, PAK6, PDPK1 |
| **TGF-beta signaling pathway** | **4 miRNAs**: hsa-miR-769-5p, hsa-miR-30e-3p, hsa-miR-493-5p, hsa-miR-181d-5p | **14 genes**: SMAD2, BMP5, PITX2, CUL1, SKP1, ACVR2A, SP1, MAPK1, CREBBP, TGFBR2, RPS6KB1 |

KEGG: Kyoto Encyclopedia of Genes and Genomes

**Supplementary Table S4. Functional enrichment analysis of differentially expressed miRNAs in LSG from SS patients with high focus score and sicca controls.**

| **Sicca controls vs SS-Patients with high focus score** | | |
| --- | --- | --- |
| **KEGG pathway** | **Examples of miRNAs** | **Examples of target mRNAs** |
| **PI3K-Akt signaling pathway** | **16 miRNAs**: hsa-miR-181d-5p, hsa-miR-30a-3p, hsa-miR-200c-3p, hsa-miR-582-5p | **130 genes**: ITGB1, ITGA8, ITGA3, TLR4, ITGA1, ITGAV, IL4R, COL5A1, COL4A3, ITGA2 |
| **MAPK signaling pathway** | **16 miRNAs**: hsa-miR-181d-5p, hsa-miR-183-5p, hsa-miR-23a-3p, hsa-miR-424-5p | **104 genes**: SOS2, MAP4K2, MAP3K3, MAPK14, MAP2K5, MAP4K3, MAP4K4, MAPK8IP1, MAP3K4, MAP3K1 |
| **Regulation of actin cytoskeleton** | **15 miRNAs**: hsa-miR-148a-5p, hsa-miR-200c-3p, hsa-miR-345-5p, hsa-miR-181d-5p | **84 genes**: ACTN2, ITGB1, EZR, ITGB8, NRAS, ITGAV, ITGA2, SOS1, ITGA10, ITGA7 |
| **Wnt signaling pathway** | **14 miRNAs**: hsa-miR-199a-5p, hsa-miR-181d-5p, hsa-miR-148a-5p, hsa-miR-30a-3p | **64 genes**: GSK3B, WNT16, FZD5, WNT7A, FZD6, WNT2B, FZD3, NFATC4, FZD4, NFATC3 |
| **Tight junction** | **14 miRNAs**: hsa-miR-24-3p, hsa-miR-148a-5p, hsa-miR-181d-5p, hsa-miR-200c-3p | **55 genes**: ACTN2, OCLN, MPP5, CLDN16, CTNNB1, PRKCB, AKT3, CTNNA3, CLDN2, PTEN, |
| **Insulin signaling pathway** | **16 miRNAs**: hsa-miR-148a-5p, hsa-miR-582-5p, hsa-miR-181d-5p, hsa-miR-424-5p | **59 genes**: CBL, CRKL, IKBKB, G6PC, SOS1, IRS1, PRKAB2, SHC4, AKT3, EIF4E2 |
| **TGF-beta signaling pathway** | **15 miRNAs**: hsa-miR-24-3p, hsa-miR-148a-5p, hsa-miR-181d-5p, hsa-miR-424-5p | **38 genes**: SMAD2, SMURF2, MYC, SMAD5, SP1, EP300, IFNG, TNF, CREBBP, TGFBR2 |
| **T cell receptor signaling pathway** | **14 miRNAs**: hsa-miR-424-5p, hsa-miR-125b-2-3p, hsa-miR-181d-5p, hsa-miR-24-3p | **54 genes**: IKBKB, NFKBIE, NFATC2, IFNG, TNF, NFATC3, CD8A, SOS1, CD28, JUN |
| **mTOR signaling pathway** | **14 miRNAs**: hsa-miR-181d-5p, hsa-miR-424-5p, hsa-miR-148a-5p, hsa-miR-23a-3p | **32 genes**: RICTOR; IKBKB; HIF1A; PIK3R1; PRKCB; AKT3; EIF4E3; VEGFA; PTEN; MAPK1 |
| **B cell receptor signaling pathway** | **14 miRNAs**: hsa-miR-424-5p, hsa-miR-199a-5p, hsa-miR-582-5p, hsa-miR-181d-5p | **24 genes**: IKBKB, PIK3R1, AKT3, MAP2K1, NFATC3, JUN, IKBKB, SOS1, NFKBIE, NRAS |
| **Protein processing in endoplasmic reticulum** | **16 miRNAs**: hsa-miR-181d-5p, hsa-miR-183-5p, hsa-miR-660-5p, hsa-miR-30a-3p | **95 genes**: ATF6, SEL1L, HSPA5, CANX, CALR, UBE2D1, DERL1, EIF2AK3, EDEM1, SEC62 |
| **N-Glycan biosynthesis** | **8 miRNAs**: hsa-miR-582-5p, hsa-miR-424-5p, hsa-miR-148a-5p, hsa-miR-199a-5p | **18 genes**: ALG13, DPAGT1, B4GALT1, ALG3, MGAT1, ALG8, MAN1A1, ST6GAL1, ALG6, MGAT2 |

KEGG: Kyoto Encyclopedia of Genes and Genomes

**Supplementary Table S5. Functional enrichment analysis of differentially expressed miRNAs in LSG from SS patients with low focus score and SS patients with high focus score.**

| **SS-Patients with low focus score vs SS-Patients with high focus score** | | |
| --- | --- | --- |
| **KEGG pathway** | **Examples of miRNAs** | **Examples of target mRNAs** |
| **Mucin type O-Glycan biosynthesis** | **16 miRNAs**: hsa-miR-181a-2-3p, hsa-miR-345-5p, hsa-miR-148a-5p, hsa-miR-320c | **16 genes**: POC1B-GALNT4, B4GALT5, GALNTL5, GCNT4, GALNT18, GALNT13, GALNT4, GALNT1, C1GALT1, GCNT1 |
| **TGF-beta signaling pathway** | **25 miRNAs**: hsa-miR-374a-5p, hsa-miR-497-5p, hsa-miR-493-5p, hsa-miR-125b-2-3p | **58 genes**: TGFBR1, ROCK1, SMAD2, MYC, TGFB2, IFNG, SMAD7, TNF, MAPK1, TGFBR2 |
| **ECM-receptor interaction** | **22 miRNAs**: hsa-miR-30a-3p, hsa-miR-381-3p, hsa-miR-125b-5p, hsa-miR-340-5p | **32 genes**: ITGA1, COL1A2, ITGA6, COL4A1, ITGA9, COL4A3, ITGA2, COL4A4, COL4A5, ITGB1 |
| **Thyroid hormone signaling pathway** | **26 miRNAs**: hsa-miR-200c-3p, hsa-miR-381-3p, hsa-miR-24-3p, hsa-miR-125b-2-3p | **71 genes**: GSK3B, PIK3R5, MED13, WNT4, MED17, GATA4, ITGAV, NCOA1, STAT1, MTOR |
| **N-Glycan biosynthesis** | **20 miRNAs**: hsa-miR-125b-5p, hsa-miR-148a-5p, hsa-miR-769-5p | **30 genes**: MAN2A2, ALG1, ALG2, ST6GAL2, ALG9, MAN1A2, B4GALT1, ST6GAL1, MGATA4A |
| **Wnt signaling pathway** | **27 miRNAs**: hsa-miR-100-5p, hsa-miR-374a-5p, hsa-miR-381-3p hsa-miR-181a-2-3p | **87 genes**: FZD7, GSK3B, WNT5A, FZD6, WNT4, WNT3, FZD8, WNT2, NFATC3, NFATC1 |
| **PI3K-Akt signaling pathway** | **27 miRNAs**: hsa-miR-125b-2-3p, hsa-miR-200c-3p, hsa-miR-493-5p, hsa-miR-320c | **178 genes**: NFKB1, IFNAR2, MCL1, IL7R, TLR4, IFNAR1, JAK2, MTOR, JAK1, IL6R |
| **mTOR signaling pathway** | **25 miRNAs**: hsa-miR-125b-2-3p, hsa-miR-409-3p, hsa-miR-181a-2-3p, hsa-miR-3074-5p | **39 genes**: RICTOR, IKBKB, PIK3CB, AKT1, PIK3CA, AKT3, MTOR, TNF, MAPK1, VEGFA |
| **Endocytosis** | **27 miRNAs**: hsa-miR-23b-3p, hsa-miR-320c, hsa-miR-381-3p, hsa-miR-181a-2-3p | **112 genes**: RAB4A, TGFBR1, SMAD2, SMAD6, CAV2, RAB5A, TRAF6, TGFB2, RAB11A |
| **Regulation of actin cytoskeleton** | **26 miRNAs**: hsa-miR-183-5p, hsa-miR-199a-3p, hsa-miR-30e-3p, hsa-miR-497-5p | **115 genes**: ACTB, ITGB1, EZR, ACTG1, ITGA3, ITGA1, ITGAV, ITGA2, ITGA10, ITGA7, |
| **cAMP signaling pathway** | **28 miRNAs**: hsa-miR-582-5p, hsa-miR-183-5p, hsa-miR-3074-5p, hsa-miR-320c | **114 genes**: NFKB1, CAMK2G, CREB5, CALM1, PIK3CB, AKT1, PIK3R1, AKT3, EP300, NFATC1 |
| **T cell receptor signaling pathway** | **25 miRNAs**: hsa-miR-409-3p, hsa-miR-374a-5p, hsa-miR-493-5p, hsa-miR-497-5p | **64 genes**: NFKB1, BCL10, IKBKB, IL4, NFKBIE, JUN, NFKBIA, IFNG, TNF, SOS1, IL10 |
| **AMPK signaling pathway** | **27 miRNAs**: hsa-miR-24-3p, hsa-miR-409-3p, hsa-miR-374a-5p, hsa-miR-582-5p | **70 genes**: PIK3CB, IGF1R, CREB1, PIK3R3, PIK3R1, IGF1, AKT3, PIK3CA, MTOR, FOXO1 |
| **MAPK signaling pathway** | **28 miRNAs**: hsa-miR-181a-2-3p, hsa-miR-125b-5p, hsa-miR-30e-3p, hsa-miR-320c | **139 genes**: TGFBR1, NFKB1, IL1R1, IKBKB, TRAF6, TGFB2, IL1A, TNF, NFATC1, TGFBR2 |
| **Protein processing in endoplasmic reticulum** | **25 miRNAs**: hsa-miR-145-5p, hsa-miR-23b-3p, hsa-miR-340-5p, hsa-miR-125b-5p | **92 genes**: ATF6, UBE2E3, UGGT1, SEL1L, HERPUD1, HSPA5, PDIA6, EDEM1, EDEM3, SEC62 |

KEGG: Kyoto Encyclopedia of Genes and Genomes

**
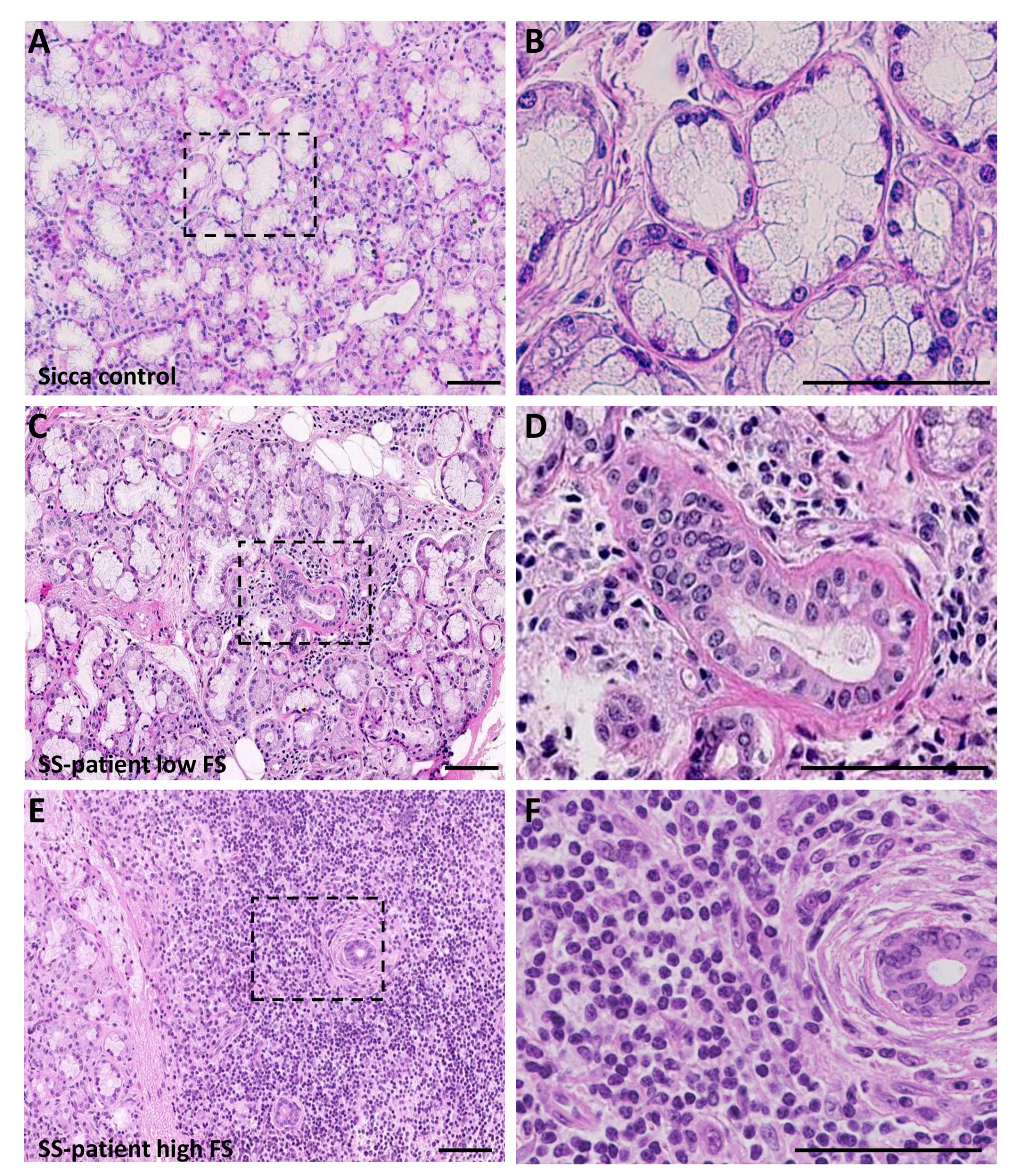
**

**Supplementary Figure S1.** Histology of LSG from a representative sicca control, SS-patients with low focus score (FS), and SS-patients with high focus score. **(A, C and E)**, Representative panoramic images of sections of LSGs stained with hematoxylin and eosin. In SS-patient with low focus score abundant quantity of parenchyma and a small inflammatory focus surrounding a duct is observed. In SS-patient with high focus score very scarce parenchyma is observed, which has been replaced by abundant inflammatory cells. **(B, D and F),** Higher magnifications of regions delimited by dashed lines in A, C, and E images. Bars: 100 μm.

**
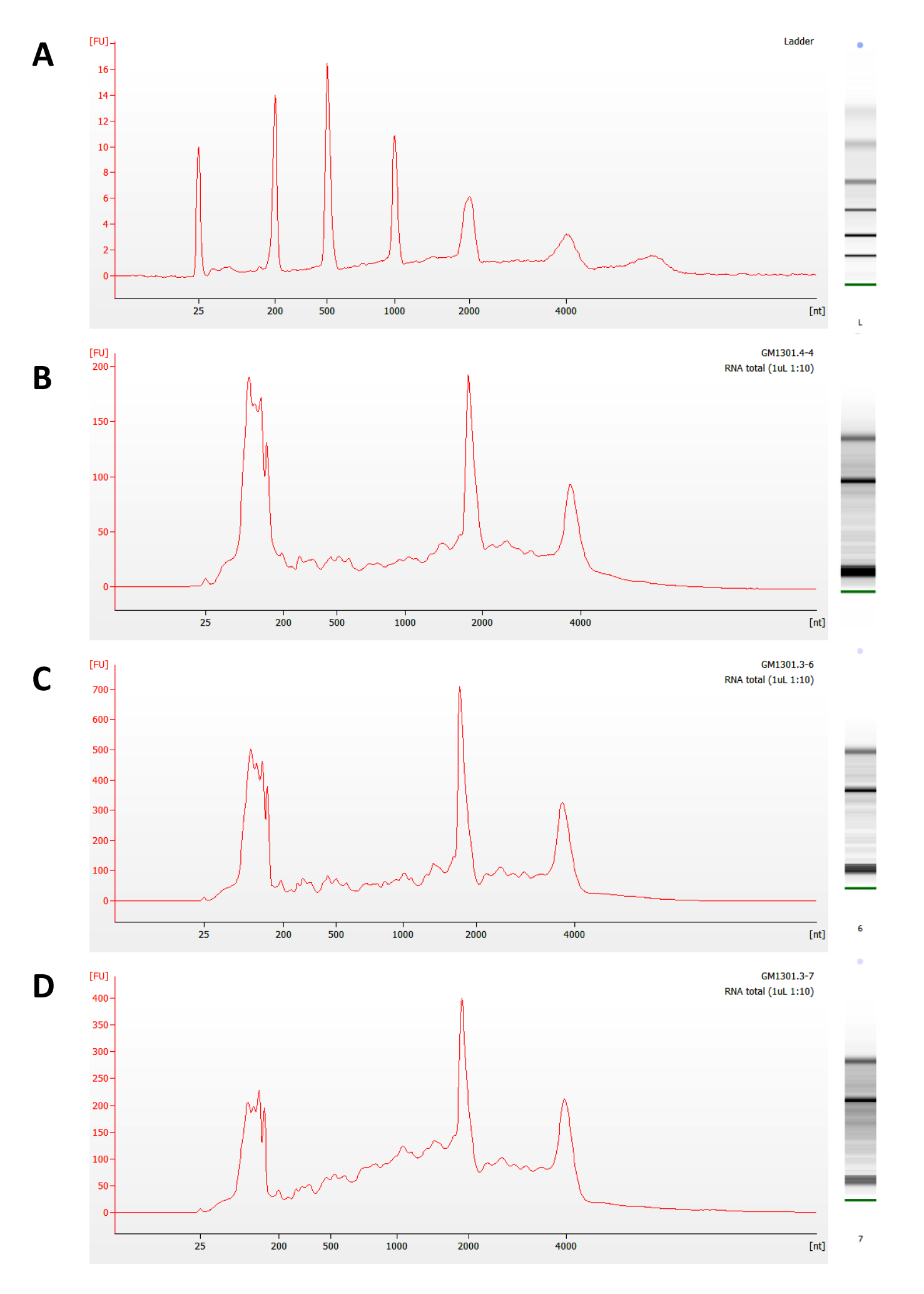
**

**Supplementary Figure S2.** Integrity of the extracted RNA. (A) Size distribution of the Agilent RNA 6000 Pico kit standard. (B) Integrity of the total RNA of a representative sample of the sicca control group. (C) Integrity of the total RNA of a sample representative of the SS-patients group with low focus score. (D) Integrity of the total RNA of a sample representative of the SS-patients group with high focus score. The samples were analyzed by Bioanalyzer using the Agilent RNA 6000 Pico kit. The peak < 200 pb corresponds to small RNAs, while the peaks of ~ 2000 and 4000 bp correspond to the ribosomal RNAs 18S and 28S, respectively.

**
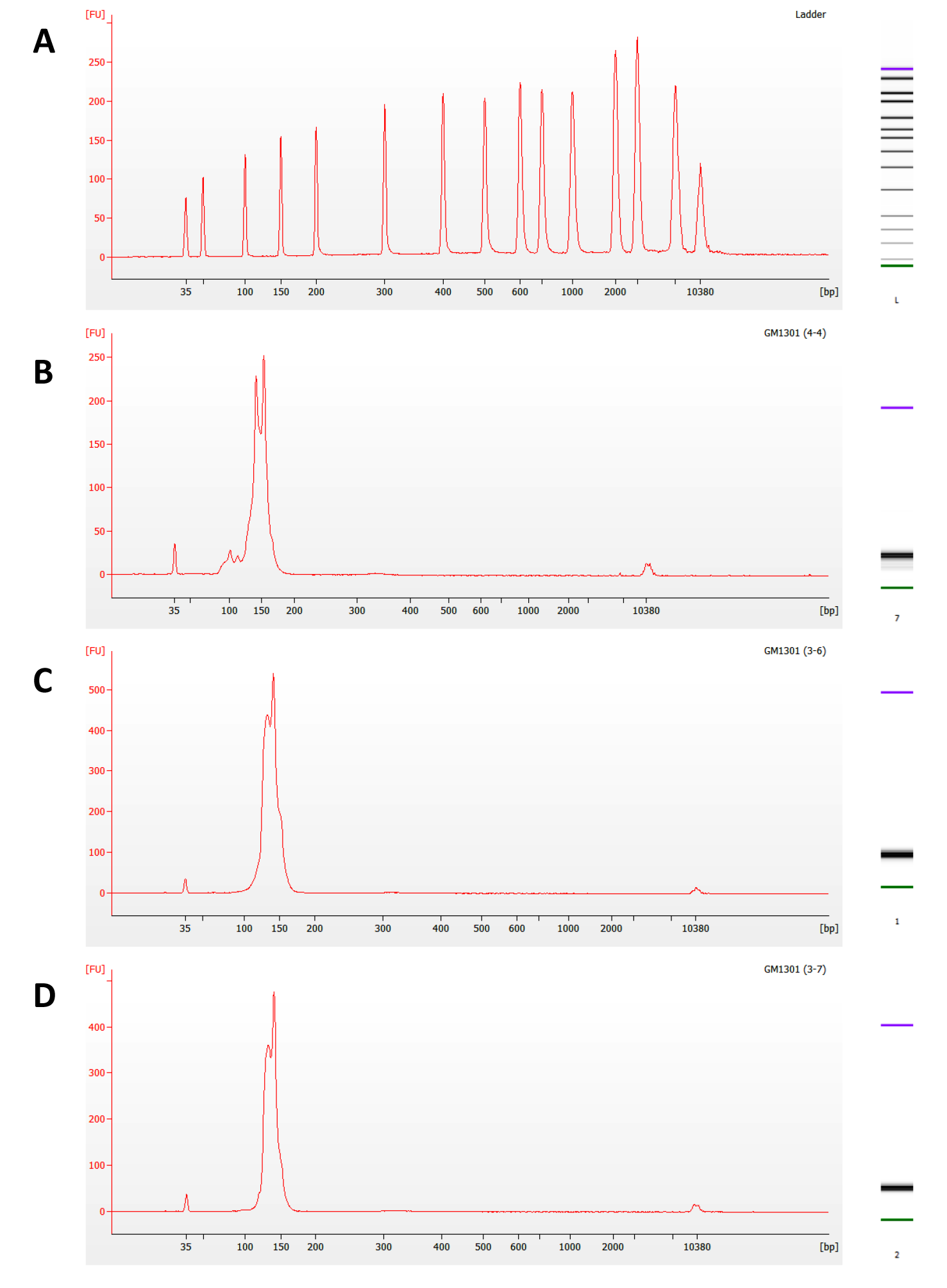
**

**Supplementary Figure S3.** Quality control of the libraries generated using the TrueSeq kit**. (A)** DNA standard of the High Sensitivity DNA kit. **(B)** Integrity of the library of a representative sample from the sicca control group, with an average fragment size of 143 bp. **(C)** Integrity of the library of a representative sample from the SS-patients group with low focus score, with an average fragment size of 137 bp. **(D)** Integrity of the library of a sample representative of the SS-patients group with high focus score, with an average fragment size of 137 bp. The samples were analyzed by Bioanalyzer.


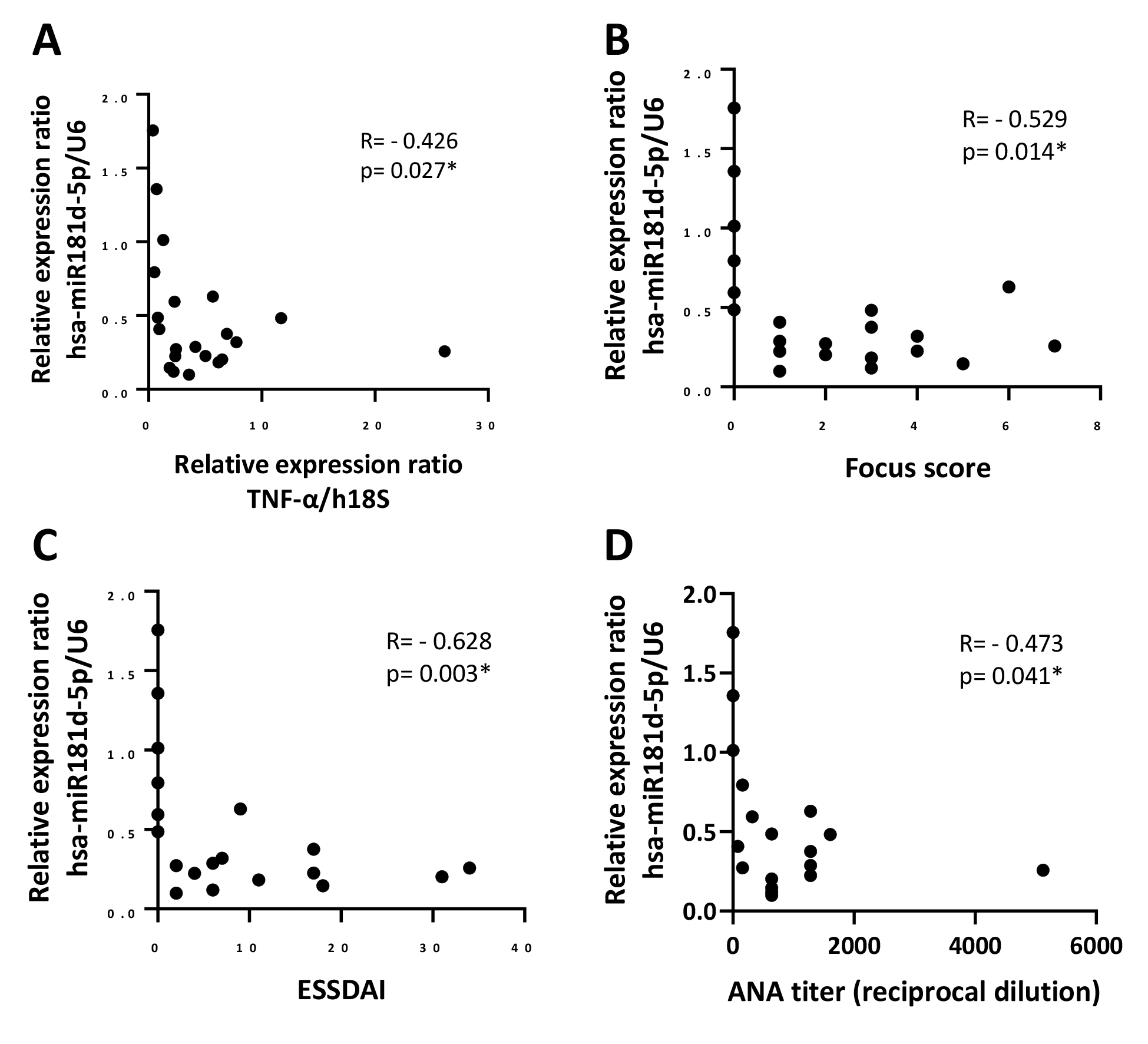


**Supplementary Figure S4. hsa-miR-181d-5p levels were inversely correlated with TNF-α, FS, ESSDAI and ANA. (A)** Spearman correlation between hsa-miR-181d-5p and TNF-α mRNA levels in SS-patients and controls. **(B)** Spearman correlation between hsa-miR-181d-5p and focus score in SS-patients and controls. **(C)** Spearman correlation between hsa-miR-181d-5p and ESSDAI in SS-patients and controls. **(D)** Spearman correlation between hsa-miR-181d-5p and ANA titers in SS-patients and controls. Data are representative of at least three independent measurements. (*) p-value ≤ 0.05 was considered significant.


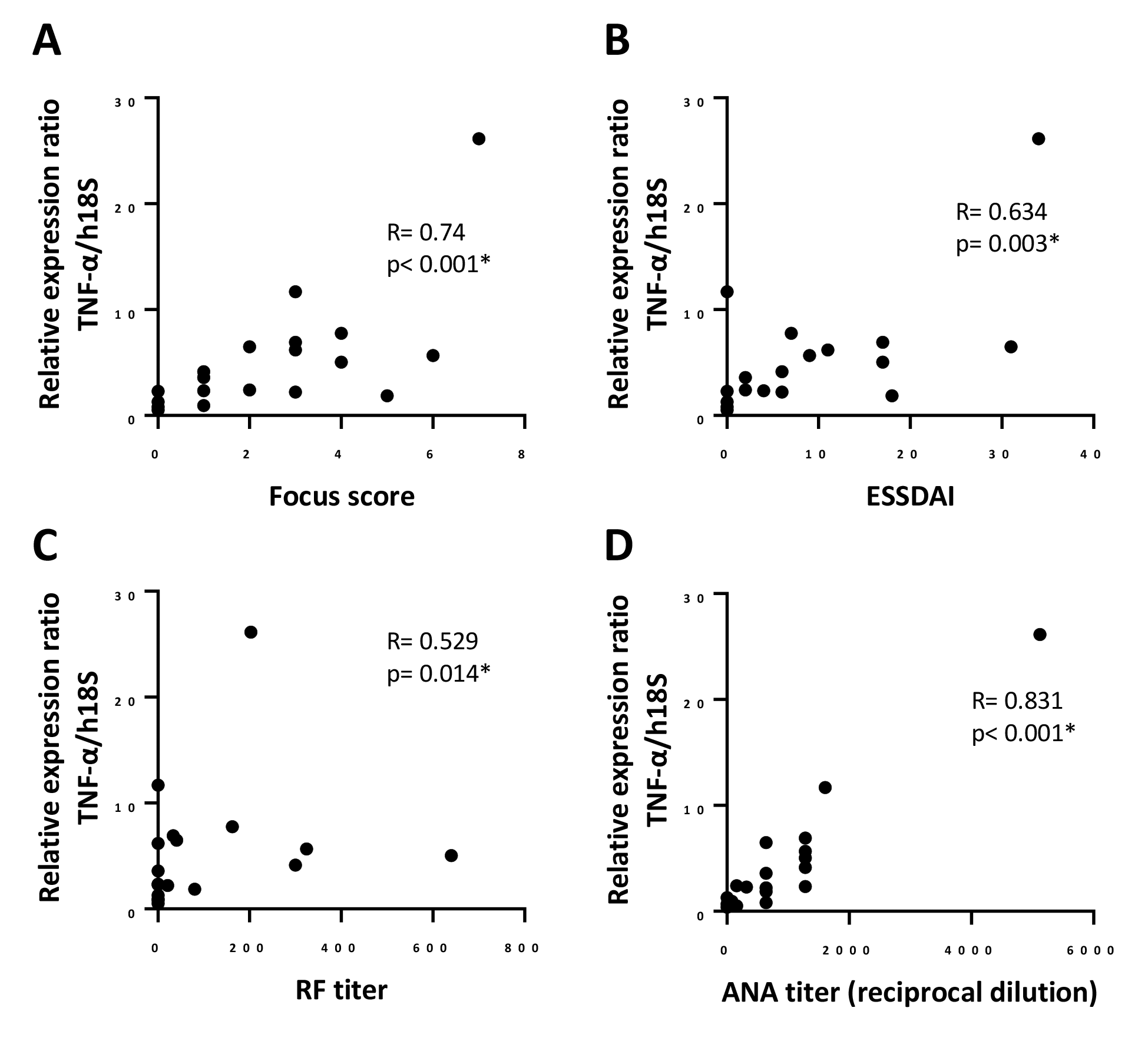


**Supplementary Figure S5. TNF-α mRNA levels were inversely correlated with FS, ESSDAI, RF and ANA. (A)** Spearman correlation between TNF-α mRNA levels and focus score in SS-patients and controls. **(B)** Spearman correlation between TNF-α mRNA levels and ESSDAI in SS-patients and controls. **(C)** Spearman correlation between TNF-α mRNA levels and RF titers in SS-patients and controls. **(D)** Spearman correlation between hsa-miR-181d-5p and ANA titers in SS-patients and controls. Data are representative of at least three independent measurements. (*) p-value ≤ 0.05 was considered significant.
